# Outcome of ICU patients with *Clostridium difficile *infection

**DOI:** 10.1186/cc11852

**Published:** 2012-11-05

**Authors:** Jean-Ralph Zahar, Carole Schwebel, Christophe Adrie, Maité Garrouste-Orgeas, Adrien Français, Aurélien Vesin, Molière Nguile-Makao, Alexis Tabah, Kevin Laupland, Alban Le-Monnier, Jean-François Timsit

**Affiliations:** 1 INSERM U823; University Grenoble 1 - Albert Bonniot Institute, Rond-point de la Chantourne, Grenoble, 38042, France; 2 Microbiology and Infection Control Unit, Necker Teaching Hospital, 145 rue de sèvres, Paris 75015, France; 3 Medical-Surgical ICU, Delafontaine Hospital, 2 rue du docteur Delafontaine, Saint-Denis, 93205, France; 4 ICU, Saint Joseph Hospital, 185 Rue Raymond Losserand, Paris, 75014, France; 5 Medical ICU, Albert Michallon Teaching Hospital, Boulevard de la Chantourne, Grenoble, 38043, France; 6 Critical Care Medicine, Peter Lougheed Centre and University of Calgary, 26th Street NE, Calgary, AB T1Y 6J4, Canada; 7 Microbiology department, André Mignot Hospital, 177 rue de Versailles, Le Chesnay, 78157, France; 8 EA 4043, USC INRA, Paris-Sud 11 University, 5 Rue Jean Baptiste Clément, Châtenay Malabry, 92290, France

## Abstract

**Introduction:**

As data from *Clostridium difficile *infection (CDI) in intensive care unit (ICU) are still scarce, our objectives were to assess the morbidity and mortality of ICU-acquired CDI.

**Methods:**

We compared patients with ICU-acquired CDI (watery or unformed stools occurring ≥ 72 hours after ICU admission with a stool sample positive for *C. difficile *toxin A or B) with two groups of controls hospitalized at the same time in the same unit. The first control group comprised patients with ICU-acquired diarrhea occurring ≥ 72 hours after ICU admission with a stool sample negative *for C. difficile *and for toxin A or B. The second group comprised patients without any diarrhea.

**Results:**

Among 5,260 patients, 512 patients developed one episode of diarrhea. Among them, 69 (13.5%) had a CDI; 10 (14.5%) of them were community-acquired, contrasting with 12 (17.4%) that were hospital-acquired and 47 (68%) that were ICU-acquired. A pseudomembranous colitis was associated in 24/47 (51%) ICU patients. The median delay between diagnosis and metronidazole administration was one day (25^th ^Quartile; 75^th ^Quartile (0; 2) days). The case-fatality rate for patients with ICU-acquired CDI was 10/47 (21.5%), as compared to 112/443 (25.3%) for patients with negative tests. Neither the crude mortality (cause specific hazard ratio; CSHR = 0.70, 95% confidence interval; CI 0.36 to 1.35, *P *= 0.3) nor the adjusted mortality to confounding variables (CSHR = 0.81, 95% CI 0.4 to 1.64, *P *= 0.6) were significantly different between CDI patients and diarrheic patients without CDI. Compared to the general ICU population, neither the crude mortality (SHR = 0.64, 95% CI 0.34 to 1.21, *P *= 0.17), nor the mortality adjusted to confounding variables (CSHR = 0.71, 95% confidence interval (CI) 0.38 to 1.35, *P *= 0.3), were significantly different between the two groups. The estimated increase in the duration of stay due to CDI was 8.0 days ± 9.3 days, (*P *= 0.4) in comparison to the diarrheic population, and 6.3 days ± 4.3 (*P *= 0.14) in comparison to the general ICU population.

**Conclusions:**

If treated early, ICU-acquired CDI is not independently associated with an increased mortality and impacts marginally the ICU length of stay.

## Introduction

Since 2000, multiple hospital-based *Clostridium difficile *infection (CDI) outbreaks have been described worldwide, and recent papers from North America have suggested an increased risk for in-hospital mortality [1,2]. However, these studies have provided conflicting results and the effects of hospital-acquired CDI on patients' outcomes remain incompletely understood. The reported mortality rates associated with *C. difficile *vary, up to 83% in some studies [3,4]. Previous studies have been inconsistent in this observation [5,6] and this may reflect different patient populations or limitations in study design, as several of these studies have failed to include a control group [7], or to include different infecting strains [1], or have not adjusted for confounding variables, such as severity of illness. Despite the fact that *C. difficile *is one of the most important causes of nosocomial infection in the intensive care unit (ICU), studies on CDI consequences are still rare. Among critically ill patients, CDI seems to be responsible for a 6% incremental increase in the risk of attributable mortality [8]. But it is difficult to determine the true attributable mortality for CDI in studies conducted on specific populations, such as the elderly or burn patients. As a result of the inherent severity of critical illness, the impact of acquisition of CDI may be expected to be greatest in ICU. Therefore, the objective of this study was to examine the influence of the development of CDI on the ICU patients' mortality.

## Materials and methods

### Study population

The present study used data in an endemic setting from three medical and/or surgical centers of the multicenter prospective cohort OUTCOMEREA™, with homogeneous procedures for microbiological diagnosis of CDI. Patients were included between January 1999 and January 2009. ICU-acquired CDI was defined as watery or unformed stools, according to the Bristol stool chart [9], in a 24-hour period occurring ≥ 72 hours after ICU admission with a laboratory confirmation of a stool sample positive for *C. difficile *toxin A or B by an immunoassay enzyme [10]. Two control groups were chosen, the first including patients hospitalized at the same time in the same unit with watery or unformed stools in a 24-hour period occurring > 72 hours after ICU admission, but with a stool sample negative for *C. difficile *toxin A or B and a negative stool culture. The second one comprised patients hospitalized at the same time and the same unit. Pseudomembranous colitis was defined as the presence confirmed by endoscopy of typical plaque-like lesions of the pseudomembrane in patients with CDI as defined above.

All codes and definitions were established prior to the study initiation. All practitioners used the same definition before any testing. Moreover, the Quality of the Database was systematically controlled. The data-capture software automatically conducted multiple checks for internal consistency of most of the variables at entry in the database. Queries generated by these checks were resolved with the source ICU before any incorporation of the new data into the database. At each participating ICU, the data quality was controlled by having a senior physician from another participating ICU checking a 2% random sample of the study data. A one-day coding course is organized annually with the study investigators and clinical research organization monitors.

The following data were collected: admission characteristics - age, sex, and origin; body weight; diagnosis at ICU admission; admission category - main reason for ICU admission; chronic diseases; McCabe score; main clinical features; and treatments used, including antimicrobials. The following scores were computed at admission, then once a day: Simplified Acute Physiologic Score (SAPSII) [11], Logistic Organ Dysfunction (LOD) [12,13], and Sequential Organ Failure Assessment (SOFA) [12,14]. Daily data about use of procedures, antibiotic consumption and proton-pump inhibitor were also collected. We recorded the durations of invasive mechanical ventilation, of the ICU and hospital stays, vital status at ICU and at hospital discharge. According to French law, this database study did not require informed consent.

### Statistical analysis

Results are expressed as frequencies and percentages for categorical variables, and as medians and quartiles for continuous variables. Independent risk factors of ICU-acquired CDI were identified using multivariate logistic regression (See Additional file 1). Patients were followed from ICU admission to the occurrence of one event, or censored at ICU discharge. Two different analyses were performed using either the overall population or only the patients with diarrhea and sampled for CDI.

In the overall population analysis, univariate risk factors of ICU death were detected using a Cause Specific Hazard model [15]. ICU admission was considered as time 0. Death in the ICU was the variable of interest, whereas discharge alive from ICU was considered as a competing event with ICU death [16]. ICU-acquired CDI was included as a time-dependent variable, which equals to 0 before infection, and to 1 from the day of CDI until the end of the follow-up. Last, a Cause Specific Hazard model was conducted to assess the impact of CDI on prognosis, with adjustment on time-fixed and time-dependent confounding factors, such as iatrogenic events occurring between admission and the CDI, bloodstream infection during ICU stay (BSI), ventilator-associated pneumonia (VAP), surgical site infection, pneumothorax during ICU stay, gastrointestinal bleeding during ICU stay, and severe hypernatremia [17,18].

In the second analysis, we only used patients with diarrhea. The time of CDI test performance was considered as Day 0, and CDI infection was considered as a time-fixed covariate. Other covariates were introduced in a Cause Specific Hazard model as previously described.

Results were presented with Cause Specific Hazard ratios (CSHRs) and 95% confidence intervals (95% CI). Models were stratified by center.

Finally, we estimated the prolongation of ICU stay using the disability model approach [19]. We used a multi-state model with four states, and all diarrheic populations started in an initial state. Then, prolongation of ICU stay was determined by reaching one of two competing absorbing states, (death or discharge alive), by taking into account the intermediate state (ICU-acquired CDI). Finally, we computed standard error estimation for prolongation of ICU stay thanks to the bootstrap method and 2,000 random samples with replacement and computed *P-*value using the Wald test. *P-*values < 0.05 were considered significant. Statistical analysis was performed using SAS 9.1 (SAS Institute, Cary, NC, USA). Length of stay prolongation was calculated with R software (R foundation, Vienna, Austria), using the change LOS library.

Assuming a 40% rate of hospital death in the diarrheic population, 471 patients were necessary to detect a hazard ratio (HR) of 2 for death with greater than 90% power and a type I error of 0.05 [20]. Similarly, 4,290 patients were necessary, assuming a 35% rate of hospital death in the whole population.

### Ethical issues

According to French law, this study did not require patient consent, as it involved research on a database. The study was approved by the institutional review board of the Centre d'Investigation Rhône-Alpes-Auvergne.

## Results

**Figure 1:**
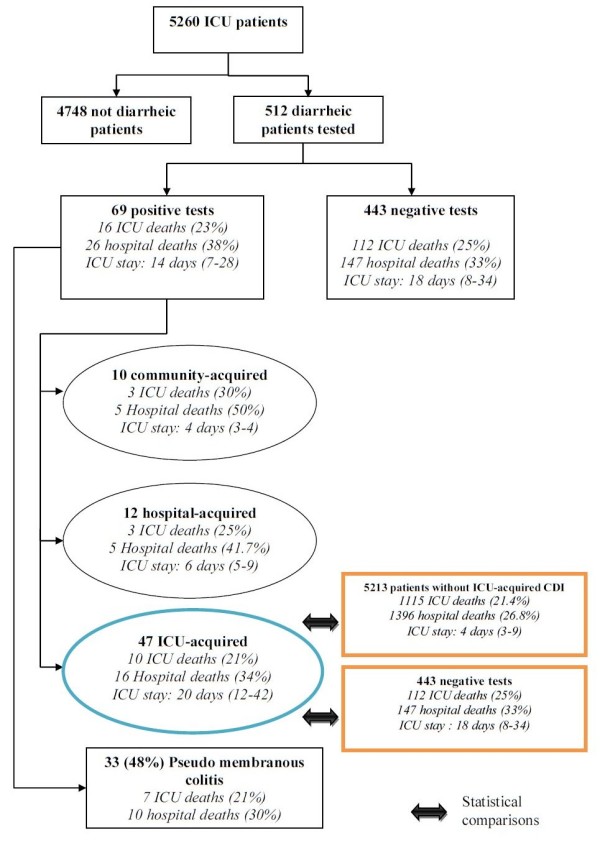
**Flow chart of patients**.

From 5,260 patients collected in the three centers, 512 patients (9.7%) underwent CD toxin testing by enzyme-linked immunosorbent assay on fecal samples for an episode of watery or unformed stools, of which 69 (69/512 = 13.5%) patients were positive. This corresponds to an incidence of ICU-acquired diarrhea of 0.97/1,000 patients-days (Figure 1).

Among the 512 patients tested, 315 (61.5%) were men, median age was 67 years (1^st ^and 3^rd ^Quartiles: 56 to 76 years) and the average SAPS and LOD were respectively 45 (1^st ^and 3^rd ^Quartiles: 36 to 59) and 6 (1^st ^and 3^rd ^Quartiles: 4 to 8). At least one chronic illness was present in 226 (44.1%) patients, and 128 (25%) patients died during ICU stay (33.8% during hospital stay). Characteristics of tested, ICU-acquired CDI patients, and non ICU-acquired CDI patients are shown in Table 1.

**Table 1:** Patients' characteristics.

Characteristics	Tested patients (*n *= 512)	Patients with ICU-acquired CDI (*n *= 47)	Patients without ICU-acquired CDI (*n *= 5,213)
Age	67 (56 to 76)	63 (55 to 75)	64 (51 to 76)
Male	315 (61.5)	29 (61.7)	3,172 (60.9)
Category of admission			
Medicine	384 (75)	38 (80.9)	4,076 (78.2)
Emergency surgery	76 (14.8)	5 (10.6)	671 (12.9)
Scheduled surgery	52 (10.2)	4 (8.5)	466 (8.9)
Main symptom at admission			
Multiple organ failure	22 (4.3)	2 (4.3)	135 (2.6)
Septic shock	110 (21.5)	12 (25.5)	575 (11.0)
Hemorrhagic shock	14 (2.7)	2 (4.3)	203 (3.9)
Cardiogenic shock	19 (3.7)	0	201 (3.9)
Mixed or other shock	11 (2.1)	1 (2.1)	114 (2.2)
Acute respiratory failure	155 (30.3)	17 (36.2)	1,191 (22.9)
Acute renal failure	34 (6.6)	0	264 (6.1)
Coma	64 (12.5)	7 (14.9)	956 (18.3)
Chronic illness			
Hepatic	40 (7.8)	7 (14.9)	326 (6.3)
Cardiovascular	60 (11.7)	7 (14.9)	675 (13.0)
Pulmonary	67 (13.1)	7 (14.9)	797 (15.3)
Renal	20 (3.9)	5 (10.6)	304 (5.8)
Immunosuppression	91 (17.8)	6 (12.8)	631 (12.1)
Diabetes mellitus	57 (11.1)	7 (14.9)	782 (15.0)
LOD	6 (4 to 8)	7 (5 to 9)	5 (3 to 7)
SOFA	8 (5.5 to 11)	8 (6 to 12)	6 (3 to 9)
SAPS II	45 (36 to 59)	50 (39 to 63)	39 (28 to 54)
Duration of Mechanical ventilation	12 (3 to 26)	14(8 to 29)	1 (0 to 6)
Duration of Proton pump inhibitor	12 (5 to 25)	15(9 to 30)	3 (0 to 7)
McCabe score			
Unplanned death in five years	292 (57)	32 (68.1)	3,047 (58.5)
Planned death between one and five years	184 (35.9)	12 (25.5)	1,697 (32.6)
Planned death in a year	36 (7)	3 (6.4)	459 (8.8)
Prognosis			
Death during ICU stay	128 (25)	10 (21.3)	1,115 (21.4)
Death during hospital stay	173 (33.8)	16 (34)	1,396 (26.8)
Duration of ICU stay	17 (8 to 33.5)	20 (12 to 42)	4 (3 to 9)
Duration of hospital stay	40 (23 to 67)	46 (28 to 78)	19 (9 to 36)

Frequencies (percentage), Median (Q1 25% to Q3 75%). CDI: *Clostridium difficile *infection; LOD, Logistic Organ Dysfunction; SAPS, Simplified Acute Physiological Score; SOFA, Systemic Organ Failure Assessment;

The case group consisted of 47 (68%) ICU-acquired CDI (incidence: 3.6/1,000 patient-days). Of these patients with CDI, 24 (51%) had a pseudomembranous colitis (incidence 1.84/1,000 patient-days). Among the 47 ICU-acquired CDI, the median time elapsed between ICU admission and first symptoms of CDI, was 8 (5 to 18) days. Three patients had septic shock at diagnosis and one required a surgical treatment. Thirty-one (66%) patients received metronidazole as first line treatment, 15 (32%) received vancomycin, and two patients received a combination therapy. Median time to initiate specific antibiotic therapy was one day (0; 2) after stool sampling. Our epidemiological data and the absence of strains resistant to fluoroquinolones suggest that none of our isolates belonged to the epidemic clone O27. Characteristics of the 47 ICU-acquired CDI patients were described in Table 2. The rate of patients tested was homogeneous across centers (data not shown).

**Table 2:** Characteristics of ICU-acquired CDI patients

Characteristics		Values
**Age**		63 (55; 75)
**Sex**	Female	18 (38.3)
	Male	29 (61.7)
**Immunocompromised**	No	41 (87.2)
	Yes	6 (12.8)
**Diabetes**	No	40 (85.1)
	Yes	7 (14.9)
**Renal chronic disease (Knaus)**	No	42 (89.4)
	Yes	5 (10.6)
**Cancer**	No	46 (97.9)
	Yes	1 (2.1)
**McCabe Score**	Unplanned death in five years	32 (68.1)
	Planned death between one and five years	12 (25.5)
	Planned death in a year	3 (6.4)
**Pseudomenbranous colitis**	No	23 (48.9)
	Yes	24 (51.1)
**Maximum wbc^$^**		13,660 (10,100; 19,400)
**Maximum creat^$^**		120 (73; 206)
**Maximum temperature (°C)**		38.5 (38; 38.8)
**Maxmimu Ht (%)**		28.5 (27; 31.5)
**Corticosteroids**	No	30 (63.8)
	Yes	17 (36.2)
**Enteral nutrition**	No	15 (31.9)
	Yes	32 (68.1)
**CDI severity score^£^**	2	1 (2.1)
	3	7 (14.9)
	4	14 (29.8)
	5	14 (29.8)
	6	7 (14.9)
	7	4 (8.5)
**SOFA score (CDI day)**		5 (3; 7)
**SOFA coagulation**		0 (0; 1)
**SOFA respiratory**		1 (1; 2)
**SOFA liver**		0 (0; 0)
**SOFA hemodynamic**		1 (0; 1)
**SOFA neurology**		1 (0; 3)
**SOFA kidney**		0 (0; 2)

$, calculated on the day of CDI and two days before. £, one point each is given for an age of > 60 years, a temperature of > 38.3 C, an albumin level of < 2.5 mg/dL, and a WBC count of > 15,000 cells/mm^3^; two points each are given for the presence of pseudomembranous colitis and hospitalization in the intensive care unit. Severe disease is considered to be present if the patient has a severity score of ≥ 2 points [28].

### Mortality

The impact of CDI on mortality was homogeneous across centers (data not shown). ICU death in patients with CDI infection was associated with a high LOD score (*P *= 0.01), a high McCabe score (*P *= 0.02), and with immunosuppression (*P *= 0.02). Two different groups were used to analyze the impact of ICU-acquired CDI on patient's outcome. The first analysis compared patients discharged alive (*n *= 4,135) versus those dead (*n *= 1,125), and showed that CDI had no significant effect on mortality as a crude (CSHR = 0.64, 95% CI 0.34 to 1.21, *P *= 0.17) or adjusted factor (CSHR = 0.71, 95% CI 0.38 to 1.35, *P *= 0.3) (See Table 3).

**Table 3:** Multivariate analysis to estimate independent prognostic effect of ICU acquired CDI in ICU patients (*n *= 5,260)

Parameter	CSHR_Death _(95%CI) *P*	CSHR_Discharge _(95%CI) *P*
**Fixed variables at admission**		
**Symptom of septic shock**	0.813 (0.68 to 9.77) *P *= 0.02	0.82 (0.73 to 0.92) *P *= 0.0005
**Symptom of acute respiratory failure**	0.898 (0.77 to 1.04) *P *= 0.16	0.61 (0.57 to 0.66) P < .0001
**Presence of at least one chronic disease**	1.04 (0.92 to 1.19) *P *= 0.5	0.91 (0.85 to 0.97) *P *= 0.005
**McCabe: death expected within five years**	1.57 (1.37 to 1.79) P < .0001	0.89 (0.83 to 0.95) *P *= 0.001
**Fixed variables in the first 48 hours**		
**Urinary bladder catheter**	0.506 (0.41 to 0.63) *P <*.0001	0.68 (0.62 to 0.74) *P *< .0001
**Vasopressors**	1.317 (1.13 to 1.54) *P *= 0.0006	0.80 (0.73 to 0.87) *P *< .0001
**Central catheter**	0.944 (0.81 to 1.10) *P *= 0.5	0.65 (0.60 to 0.70) *P *< .0001
**Mechanical ventilation**	1.037 (0.86 to 1.25) *P *= 0.7	0.70 (0.65 to 0.76) *P *< .0001
**DNR order**	3.146 (2.72 to 3.64) P < .0001	0.56 (0.47 to 0.66) *P *< .0001
**SAPS II : ≤ 36 pts**	1; *P *< .0001	1; *P *< .0001
**37 to 45**	1.90 (1.48 to 2.44)	0.81 (0.74 to 0.88)
**46 to 59**	2.926 (2.30 to 3.72)	0.74 (0.68 to 0.82)
**≥ 60**	7.715 (6.09 to 9.77)	0.48 (0.42 to 0.55)
**Time dependant variables**		
**CRBSI**	1.67 (1.12 to 2.48) *P *= 0.01	0.97 (0.67 to 1.41) *P *= 0.9
**Other BSI**	1.08 (0.87 to 1.35) *P *= 0.5	0.45 (0.39 to 0.54) *P *< .0001
**VAP without BSI**	1.00 (0.82 to 1.22) *P *= 1	0.47 (0.41 to 0.54) *P *< .0001
**Deep and organ/space surgical site infection without BSI**	0.79 (0.38 to 1.65) *P *= 0.5	0.76 (0.53 to 1.09) *P *= 0.14
**Pneumothorax**	1.03 (0.73 to 1.47) *P *= 0.9	0.47 (0.35 to 0.64) *P *< .0001
**Gastrointestinal bleeding**	1.82 (1.27 to 2.61) *P *= 0.001	0.79 (0.54 to 1.14) *P *= 0.21
**Severe hypernatremia**	1.67 (1.37 to 2.04) *P *< .0001	0.81 (0.70 to 0.94) *P *= 0.005
**CDI**	**0.71 (0.38 **to **1.35) *P *= 0.3**	**0.74 (0.52 **to **1.06) *P *= 0.097**

BSI, Blood Stream Infection; CDI, *Clostridium difficile *infection; CRBSI, Catheter-Related Blood Stream Infection; CSHR (95% CI), Cause Specific Hazard Ratio and 95% confidence interval; DNR, Do Not Resuscitate; SAPS, Simplified Acute Physiological Score. Other variables that were tested but not retained in the model were the following, at admission, age, sex, category of admission, AVC diagnosis, AIDS and corticosteroid use; in the first 48 hours, Proton Pump Inhibitor and SOFA. See Additional file 1 for details on univariate analysis. Time dependent variables were forced in the model.

The second analysis compared ICU-acquired CDI patients with diarrheic patients with negative stool culture: the crude effect of CDI on mortality was still not significant (CSHR = 0.70, 95% CI 0.36 to 1.35, *P *= 0.3). Moreover, after adjustment on confounding factors and iatrogenic events between admission and occurrence of diarrhoea, the effect on mortality remained not significant (CSHR = 0.81, 95% CI 0.40 to 1.64, *P *= 0.6) (See Table 4).

**Table 4:** Multivariate analysis to estimate independent prognostic effect of ICU-acquired CDI in the tested diarrheic patients (*n *= 490)

Parameter	CSHR_Death _(95% CI) *P *	CSHR_Discharge _(95% CI) *P*
**Fixed variables at admission**		
**CVA diagnosis at admission**	3.94 (1.55 to 10.0) *P *= 0.004	1.19 (0.61 to 2.32) *P *= 0.6
**Cardiac chronic disease (Knaus)**	1.78 (1.09 to 2.9) *P *= 0.02	0.96 (0.67 to 1.38) *P *= 0.8
**AIDS**	2.91 (0.88 to 9.67) *P *= 0.08	0.87 (0.35 to 2.13) *P *= 0.8
**Corticosteroid use**	1.78 (0.87 to 3.65) *P *= 0.11	0.67 (0.41 to 1.10) *P *= 0.12
**Death expected (McCabe)**	1.76 (1.2 to 2.59) *P *= 0.004	1.04 (0.84 to 1.30) *P *= 0.7
**Fixed variables in the first 48 hours**		
**DNR Order**	3.01 (1.74 to 5.22) *P *< .0001	0.77 (0.47 to 1.27) *P *= 0.3
**Variable the days before test**		
**SOFA the days before CD test**	1.15 (1.09 to 1.22) *P *< .0001	0.90 (0.87 to 0.93) *P *< .0001
**Variable on the day of the test**		
**CDI**	**0.81 (0.40 **to **1.64) *P *= 0.6**	**0.70 (0.5 **to **1.01) *P *= 0.06**

AIDS, Acquired Immune Deficiency Syndrome; BSI, Blood Stream Infection; CDI, *Clostridium difficile *infection; CRBSI, Catheter Related Blood Stream Infection; DNR, Do Not Resuscitate; CSHR (95%CI), Cause Specific Hazard ratio and 95% confidence interval; CVA, Cerebrovascular accident; SAPS, Simplified Acute Physiological Score; SOFA, Sequential Organ Failure Assessment. Other variables that were tested but not retained in the model were the following: at admission, age, sex, category of admission, symptom of septic shock, symptom of acute respiratory failure, hepatic chronic disease, pulmonary chronic disease, renal chronic disease, immunosuppressive chronic disease, diabetes mellitus, presence of at least one chronic disease, AIDS, and corticosteroid use; in the first 48 hours - vasopressors, central catheter, urinary tract, mechanical ventilation, Proton Pump Inhibitor, SOFA and SAPS II. See Additional file 1 for details on univariate analysis.

Results remained similar when excluding patients with metronidazole (IV or oral) or vancomycin (oral) before the diagnostic test (adjusted CSHR = 0.84, 95% CI 0.41 to 1.71, *P *= 0.6), or when only taking into account cases with pseudomembranous colitis acquired in ICU (*n *= 24 cases, CSHR = 0.80, 95% CI 0.31 to 2.03, *P *= 0.6).

Results would only be slightly different if the 95/443 (21.4%) given metronidazole or vancomycin within 48 hours following a negative test were excluded from the statistical analysis. (adjusted CSHR_Death _= 0.80 (0.39 to 1.65), *P *= 0.5, CSHR_discharge _= 0.68 (0.46 to 1.00), *P *= 0.0475).

### Length of stay

The median length of ICU stay in the whole population of diarrheic patients was 17 (8 to 34) days, whereas the median length of ICU stay in the CDI population was 20 (12 to 42) days. Using a multistate model, the estimated prolongation of ICU stay for the diarrheic population due to *C. difficile *was 8.0 days ± 9.3 days, *P *= 0.4.

Moreover, the median length of ICU stay in the whole population was 4 (3 to 9) days, whereas the median length of ICU stay in the ICU-acquired CDI group was 20 (12 to 42) days. The estimated prolongation of ICU stay due to *C. difficile *was 6.3 days ± 4.3, *P *= 0.14

## Discussion

In our retrospective study conducted in an ICU cohort population, we found that ICU- and hospital crude mortality of CDI patients were 21 and 34%, respectively. Despite a significantly higher crude mortality, when using modern statistical models, CDI was not associated with increased mortality, regardless of the control groups, and after careful adjustment on confounding factors of mortality and on other adverse events and nosocomial infections associated with mortality.

The crude mortality rate associated with ICU-acquired CDI that we observed is similar to that observed in previous studies conducted elsewhere [7,8,21-23]. It is also notable that, even if the duration of ICU stay of CDI patients was considerably longer than that of other mechanically ventilated patients, the extra-length of stay that we estimated using a multistate model was 6.3 days and did not reach a statistical significance (*P *= 0.14).

Treatment of CDI occurred after a median delay of one day after diagnostic test sampling. The early treatment of patients probably explains the lack of significant impact on mortality.

Our results are in contradiction with previous studies conducted in ICUs that have found a higher mortality of patients with ICU-acquired CDI. Ang *et al. *found a higher crude ICU mortality of 33.9% in ICU acquired CDI as compared to other ICU patients (29%) [7]. Using a matched case-control design, Kenneally *et al. *[8] found the overall 30-day mortality rate in a cohort of 278 ICU patients with CDI equaled 36.7%, giving a 6.1% (95% CI, -1.7% to 13.9%, *P *= 0.127) CDI-attributable mortality rate. However, they did not adjust for confounding variables, such as severity of disease or other adverse events. Another study reported by Lawrence *et al. *[21] identified 40 ICU-acquired CDI in a 19-bed medical ICU during a 30-month period. Using univariate analysis, CDI neither influenced ICU- (CDI 18 vs. other 20%) nor hospital mortality (CDI 30% vs. other 28%), but was associated with an increase in the crude length of ICU- (CDI 15 days vs. other 3 days, *P *< 0.001) and hospital stay (CDI 38 vs. other 10 days, *P *< 0.001). After adjustment for severity of the acute illness, vancomycin resistant enterococcus (VRE) colonization, receipt of antimicrobial and occurrence of nosocomial infection, but without taking into account ICU time before CDI acquisition, CDI was associated with a longer ICU length of stay (OR, 1.24 (95% CI, 1.07 to 1.44)).

There are a number of potential reasons why studies have shown variable association with CDI and mortality.

### Selection of CDI patients and controls

First, the choice of control groups may influence findings, as this has been outlined in a number of epidemiological publications [22,23]. Our methodological approach was to minimize bias due to the characteristics of the control group by comparing patients with ICU-CDI, to patients with diarrhea not linked to *C. difficile*, and to the whole ICU population. Indeed, there is much potential selection bias that arises if we choose only patients with diarrhea as a control group. On the other hand, controls should be selected from the same source population or study base that gives rise to the cases. The patients whose stools have been sampled are possibly different from the ones that have not been sampled.

However, the variability of the patient populations might also explain the variability in the association between mortality and CDI disease in the patient populations under study. Our study population included all ICU patients, and was different from that of other studies that were interested in specific selected populations, such as older persons, ill patients or burn unit patients.

Finally, our epidemiological situation is different from North America's, as none of our patients had been infected with NAP1/O27 isolates. As this strain seems to be more virulent comparatively to others, our lower mortality rate could be explained by this microbiological difference. Indeed, in recent years with the emergence of a hypervirulent strain, the annual frequency of and the case fatality due to CDI have doubled in the United States [2,24,25]. Moreover, authors [1] demonstrated a higher mortality rate among inpatients in which nosocomial CDI developed compared to control subjects without CDI, matched for sex, age and disease severity; but this attributable mortality was measured during the CDI epidemic in Quebec caused by the hypervirulent strain NAP1/O27. Finally, the antimicrobial treatment was instituted early in CDI patients and may have decreased the impact of CDI on mortality and length of stay.

### Adjustment on confounders

A second consideration that may explain differences in findings among studies conducted to date is in the analysis with adequate adjustment for confounding variables and competing events for mortality. Failure to adequately adjust for factors differently distributed among patients with or without CDI that also affect their outcome may lead to different conclusions. A number of factors could explain mortality in the ICU, such as advanced age and severity of illness at onset, and the presence of sepsis or septic shock. We used a modern statistical model that is frequently applied in other medical fields, such as cancer epidemiology. This approach is based on event histories, model time-to-event and may focus on time-dependent risk factors, such as nosocomial infections. Modern statistical methods are further able to simultaneously analyze different endpoint types, and they explicitly account for the timing of events [16]. Indeed, a case-control study could have led to different findings. It is important to underline that nosocomial infection is a time-dependent event. Occurrence of nosocomial infection is a time-dynamic process, and the discharge acts as a competing risk when estimating the relationship between nosocomial infection and death. Both factors may bias the attributable mortality estimate. Matching patients with and without CDI infection on ICU duration and then performing conditional logistic regression is a widely used method to evaluate nosocomial infection (CDI here). The attributable mortality method is also used for other events that are dependent on the duration of exposure to a risk factor. With this method, each patient is classified as being exposed to CDI or unexposed (no CDI). In exposed patients, the data are handled as if the exposure was present at the study initiation (although exposure status is determined at study completion). Thus, the excess risk of death associated with the exposure is assumed to be present throughout the ICU stay, that is, both before and after the occurrence of the exposure. In other words, the exposure is handled as a time-independent variable. If the exposure is actually time-dependent, then a bias is introduced. Therefore, the impact of a time-dependent exposure on mortality is overestimated with this method. Our statistical model, in contrast, considers that the excess risk of death associated with the exposure exists only after the exposure onset. In this multistate model, each patient goes through two or more states. Thus, at study initiation, all patients are classified as being in the unexposed state. Over time, some patients acquire the exposure of interest (here, CDI); therefore switching to the exposed state, at different time points during the ICU stay. Eventually, the model fits reality far more closely than does the matched cohort design, resulting in the narrowest confidence intervals. The main advantage of using the multistate model for complete data is that mortality can be estimated over time. Therefore, the changes in the mortality rate over time can be detected.

We previously showed that about a quarter of ICU patients experienced more than one adverse event, and that nosocomial infections, such as ventilator-associated pneumonia, ICU-acquired bloodstream infections, deep and organ/space surgical site infection without BSI, and adverse events, such as pneumothorax, gastrointestinal bleeding [18] and hypernatremia [17], were independently associated with mortality. The multistate model we used allows us to avoid the estimation bias associated to these events [16,26].

### Information bias

Procedure for *C. difficile *detection is clearly defined in all study centers, and is only used in cases of watery stools. The toxin assay we used possesses an excellent specificity, but an 80% sensitivity [27]. It is, therefore, possible that some patients may have been falsely classified as belonging to the diarrheic CDI-negative control group, and thus decreased the study power (that is, the probability to find a difference if it exists). However, in the diarrheic CDI negative patients, no instance of hospital-acquired CDI was diagnosed after ICU discharge. Finally, our study was conducted in three French ICUs (in Grenoble and the Paris region), so our results cannot be extrapolated to the whole of France.

## Conclusions

This study was conducted using a large database of ICU patients in a country where hypervirulent strains are rare. After careful adjustment for confounding variables, CDI is not associated with significant attributable mortality and extra length of stay.

## Key messages

• If treated promptly, ICU-acquired CDI is not independently associated with an increased mortality, and impacts marginally the ICU-length of stay.

• Careful adjustment on confounding factors of mortality and on other adverse events is instrumental to analyzing outcomes of ICU-acquired infections.

## Abbreviations

BSI: bloodstream infection during ICU stay; CDI: *Clostridium difficile *infection; CSHR: cause specific hazard ratio; D: Death; DC: Discharge; HR: hazard ratio; LOD: logistic organ dysfunction; SAPS: simplified acute physiologic score; SOFA: sequential organ failure assessment; VAP: ventilator-associated pneumonia; VRE: vancomycin resistant enterococcus.

## Competing interests

The authors declare no competing interests.

## Authors' contributions

JRZ, AV, AF and JFT conceived the study, designed the analysis and interpretation of the data, and drafted the manuscript. JFT, AV, AF, CS, CA, MGA, MNM and AT acquired data. CS, CA, MGA, MNM, AT, KL and ALM helped with acquisition of data, critical revision of the manuscript and final approval. All authors have read and approved the manuscript for publication.

## Supplementary Material

Additional file 1 **Univariate factors associated with prognosis in ICU patient and diarrheic patients tested**. Tables with variables associated with death or discharge by univariate analysis in ICU patients and diarrheic patients tested. Click here for file
