# Supplementary material for: Outcome of ICU patients with Clostridium difficile infection
Source: Crit Care. 2012 Nov 5;16(6):R215. doi: 10.1186/cc11852 (PMC3672590; doi:10.1186/cc11852)
Supplement: Additional file 1 — Univariate factors associated with prognosis in ICU patient and diarrheic patients tested. Tables with variables associated with death or discharge by univariate analysis in ICU patients and diarrheic patients tested. [file cc11852-S1.DOC]

**Outcome of ICU patients with *Clostridium difficile* infection: Supplement material**

**Additional file 1, Table S1 :** Univariate factors associated to prognosis in ICU patient (n=5260)

| Variable | items | Discharge    (n=4135) | Death  (n=1125) | P value  Death | P value  Discharge |
| --- | --- | --- | --- | --- | --- |
| Fixed variables at admission |  |  |  |  |  |
| Age | 0 - 49 | 1099(88.7) | 140(11.3) | <.0001 | <.0001 |
|  | 50 - 64 | 1184(80.9) | 279(19.1) |  |  |
|  | 65 - 74 | 806(73.9) | 284(26.1) |  |  |
|  | ≥ 75 | 1046(71.3) | 422(28.7) |  |  |
| Male |  | 2503(78.2) | 698(21.8) | 0.3 | 0.001 |
| Category of admission | Medical | 3223(78.3) | 891(21.7) | 0.0005 | <.0001 |
|  | Emergency surgery | 520(76.9) | 156(23.1) |  |  |
|  | Scheduled surgery | 392(83.4) | 78(16.6) |  |  |
| Symptom of septic shock |  | 405(69) | 182(31) | 0.15 | <.0001 |
| Symptom of acute respiratory failure |  | 963(79.7) | 245(20.3) | <.0001 | <.0001 |
| CVA diagnosis at admission |  | 135(51.7) | 126(48.3) | <.0001 | <.0001 |
| Cardiac chronic disease |  | 469(68.8) | 213(31.2) | <.0001 | 0.19 |
| Hepatic chronic disease |  | 243(73) | 90(27) | 0.07 | 0.007 |
| Pulmonary chronic disease |  | 611(76) | 193(24) | 0.9 | 0.0007 |
| Renal chronic disease |  | 244(79) | 65(21) | 0.8 | 0.05 |
| Immunosuppressive chronic disease |  | 446(72.2) | 172(27.8) | <.0001 | 0.10 |
| Diabetes mellitus |  | 600(76) | 189(24) | 0.02 | 0.8 |
| Presence of at least one chronic disease |  | 1620(73.7) | 579(26.3) | <.0001 | <.0001 |
| AIDS |  | 50(78.1) | 14(21.9) | 0.5 | 0.4 |
| Corticosteroids use |  | 160(71.7) | 63(28.3) | 0.008 | 0.098 |
| Mc Cabe : Death expected within 5 years |  | 1474(67.9) | 697(32.1) | <.0001 | <.0001 |
| Fixed variables in the first 48h |  |  |  |  |  |
| Vasopressors |  | 1268(62.6) | 759(37.4) | <.0001 | <.0001 |
| Central catheter |  | 1662(68.8) | 755(31.2) | <.0001 | <.0001 |
| Urinary bladder catheter |  | 3126(75.7) | 1004(24.3) | 0.015 | <.0001 |
| Mechanical ventilation |  | 1904(67.9) | 900(32.1) | <.0001 | <.0001 |
| Proton pump Inhibitor |  | 2666(78.6) | 725(21.4) | 0.0002 | <.0001 |
| SOFA | 1,2,3 | 1275(93.5) | 88(6.5) | <.0001 | <.0001 |
|  | 4,5,6 | 1433(89) | 178(11) |  |  |
|  | 7,8,9 | 874(74.1) | 306(25.9) |  |  |
|  | > 9 | 553(50) | 553(50) |  |  |
| SAPS II | ≤ 36 | 2198(94.8) | 121(5.2) | <.0001 | <.0001 |
|  | 37 – 45 | 826(85.2) | 143(14.8) |  |  |
|  | 46 – 59 | 742(74.6) | 253(25.4) |  |  |
|  | > 59 | 369(37.8) | 608(62.2) |  |  |
| DNR Order |  | 135(31.3) | 296(68.7) | <.0001 | <.0001 |
| Time dependant variables |  |  |  |  |  |
| Severe hypernatremia |  | 214(59.8) | 144(40.2) | <.0001 | <.0001 |
| Pneumothorax |  | 44(55) | 36(45) | 0.08 | <.0001 |
| Deep and organ/space surgical site infection without BSI |  | 31(79.5) | 8(20.5) | 0.20 | 0.15 |
| VAP without BSI |  | 333(67.5) | 160(32.5) | 0.12 | <.0001 |
| Other BSI |  | 173(59.2) | 119(40.8) | 0.11 | <.0001 |
| CRBSI |  | 30(51.7) | 28(48.3) | 0.0001 | 0.043 |
| Gastrointestinal Bleeding |  | 30(44.8) | 37(55.2) | <.0001 | 0.0001 |
| CDI |  | 37(78.7) | 10(21.3) | 0.17 | 0.025 |

CDI: *Clostridium difficile* infection; CRBSI : Catheter-Related Blood Stream Infection ; BSI : Blood Stream Infection ; DNR : Do Not Resuscitate ; SOFA : Sequential Organ Failure Assessment ; SAPS : Simplified Acute Physiological Score ; AIDS : Acquired Immune Deficiency Syndrom ; CVA : Cerebrovascular accident

Additional file 1, **Table S2 :** Univariate factors associated to prognosis in diarrheic patients tested (n=490)

| Variable | items | Discharge  (n=368) | Death  (n=122) | P value  Death | P value  Discharge |
| --- | --- | --- | --- | --- | --- |
| Fixed variables at admission |  |  |  |  |  |
| Age | 0 - 49 | 64(86.5) | 10(13.5) | 0.005 | 0.23 |
|  | 50 - 64 | 119(82.1) | 26(17.9) |  |  |
|  | 65 - 74 | 91(70.5) | 38(29.5) |  |  |
|  | ≥ 75 | 94(66.2) | 48(33.8) |  |  |
| Male |  | 229(75.6) | 74(24.4) | 0.4 | 0.9 |
| Category of admission | Medical | 277(75.7) | 89(24.3) | 0.6 | 0.4 |
|  | Emergency surgery | 54(74) | 19(26) |  |  |
|  | Scheduled surgery | 37(72.5) | 14(27.5) |  |  |
| Symptom of septic shock |  | 71(68.3) | 33(31.7) | 0.33 | 0.4 |
| Symptom of acute respiratory failure |  | 112(74.7) | 38(25.3) | 0.6 | 0.048 |
| CVA diagnosis at admission |  | 9(64.3) | 5(35.7) | 0.035 | 0.20 |
| Cardiac chronic disease |  | 35(60.3) | 23(39.7) | 0.018 | 0.80 |
| Hepatic chronic disease |  | 25(65.8) | 13(34.2) | 0.001 | 0.46 |
| Pulmonary chronic disease |  | 47(71.2) | 19(28.8) | 0.8 | 0.8 |
| Renal chronic disease |  | 16(80) | 4(20) | 0.6 | 0.8 |
| Immunosuppressive chronic disease |  | 51(68) | 24(32) | 0.035 | 0.6 |
| Diabetes mellitus |  | 42(80.8) | 10(19.2) | 0.4 | 0.9 |
| Presence of at least one chronic disease |  | 147(68.4) | 68(31.6) | 0.0007 | 0.7 |
| AIDS |  | 5(62.5) | 3(37.5) | 0.3 | 0.8 |
| Corticosteroids use |  | 18(64.3) | 10(35.7) | 0.56 | 0.08 |
| Mc Cabe : Death expected within 5 years |  | 142(67.3) | 69(32.7) | <.0001 | 0.9 |
| Fixed variables in the first 48 hours |  |  |  |  |  |
| Vasopressors |  | 210(70.9) | 86(29.1) | 0.11 | 0.03 |
| Central catheter |  | 250(74.4) | 86(25.6) | 0.9 | 0.008 |
| Urinary bladder catheter |  | 335(74.8) | 113(25.2) | 1 | 0.038 |
| Mechanical ventilation |  | 246(72.8) | 92(27.2) | 0.8 | 0.0003 |
| Proton pump Inhibitor |  | 274(74.1) | 96(25.9) | 0.08 | 0.8 |
| SOFA | 1,2,3 | 41(85.4) | 7(14.6) | 0.3 | 0.12 |
|  | 4,5,6 | 99(77.3) | 29(22.7) |  |  |
|  | 7,8,9 | 105(72.9) | 39(27.1) |  |  |
|  | > 9 | 123(72.4) | 47(27.6) |  |  |
| SAPS II | ≤ 36 | 112(79.4) | 29(20.6) | 0.04 | 0.06 |
|  | 37 – 45 | 90(81.1) | 21(18.9) |  |  |
|  | 46 – 59 | 90(72.6) | 34(27.4) |  |  |
|  | > 59 | 76(66.7) | 38(33.3) |  |  |
| DNR Order |  | 17(50) | 17(50) | <.0001 | 0.4 |
| Variables the days before test |  |  |  |  |  |
| SOFA | 1,2,3 | 41(85.4) | 7(14.6) | <.0001 | <.0001 |
|  | 4,5,6 | 99(77.3) | 29(22.7) |  |  |
|  | 7,8,9 | 105(72.9) | 39(27.1) |  |  |
|  | > 9 | 123(72.4) | 47(27.6) |  |  |
| Variable on the day of test |  |  |  |  |  |
| CDI |  | 37 (78.7) | 10(21.3) | 0.3 | 0.09 |

CDI: *Clostridium difficile* infection; CRBSI : Catheter-Related Blood Stream Infection ; BSI : Blood Stream Infection ; DNR : Do Not Resuscitate ; SOFA : Sequential Organ Failure Assessment ; SAPS : Simplified Acute Physiological Score ; AIDS : Acquired Immune Deficiency Syndrom ; CVA : Cerebrovascular accident
